# Study on Cytotoxic and Genotoxic Potential of Bulgarian *Rosa damascena* Mill. and *Rosa alba* L. Hydrosols—*In Vivo* and *In Vitro*

**DOI:** 10.3390/life12091452

**Published:** 2022-09-19

**Authors:** Tsvetelina Gerasimova, Gabriele Jovtchev, Svetla Gateva, Margarita Topashka-Ancheva, Alexander Stankov, Tsveta Angelova, Ana Dobreva, Milka Mileva

**Affiliations:** 1 Institute of Biodiversity and Ecosystem Research, Bulgarian Academy of Sciences, 2 Gagarin Str., 1113 Sofia, Bulgaria; 2 Institute for Roses and Aromatic Plants, Agricultural Academy, 49 Osvobojdenie Blvd., 6100 Kazanlak, Bulgaria; 3 The Stephan Angeloff Institute of Microbiology, Bulgarian Academy of Sciences, 26 Acad. G. Bonchev Str., 1113 Sofia, Bulgaria

**Keywords:** *Rosa alba* L. and *Rosa damascena* Mill. hydrosols, cytotoxic and genotoxic potential, test systems *in vivo* and *in vitro*, chromosome aberrations, micronuclei, mitotic index and nuclei division index

## Abstract

The *Rosa alba* L. and *Rosa damascena* Mill. growing in Bulgaria are known for their extremely fine essential oil and valuable hydrosols. Irrespectively of its wide use in human life, little research exists on the cytotoxic and genotoxic activity of the hydrosols. This set our goal to conduct cytogenetic analyses to study these effects. A complex of classical cytogenetic methods was applied in three types of experimental test systems—higher plant *in vivo*, ICR mice *in vivo*, and human lymphocytes *in vitro*. Mitotic index, PCE/(PCE + NCE) ratio, and nuclear division index were used as endpoints for cytotoxicity and for genotoxicity—induction of chromosome aberrations and micronuclei. Rose hydrosol treatments range in concentrations from 6% to 20%. It was obtained that both hydrosols did not show considerable cytotoxic and genotoxic effects. These effects depend on the type of the tested rose hydrosols, the concentrations applied in the experiments, and the sensitivity and specificity of the test systems used. Human lymphocytes *in vitro* were the most sensitive to hydrosols, followed by higher plant and animal cells. Chromosomal aberrations and micronucleus assays suggested that *R. damascena* and *R. alba* hydrosols at applied concentrations possess low genotoxic risk. Due to the overall low values in terms of cytotoxic and/or genotoxic effects in all test systems, hydrosols are promising for further use in various areas of human life.

## 1. Introduction

Rose hydrosol is an abundantly represented product obtained by water–steam distillation of essential rose oil, the most famous of which are *Rosa damascena* Mill. and *Rosa alba* L. These hydrosols find wide spread applications in cosmetics, perfumery, pharmacy, phytotherapy, the food industry, and many other areas of human life [1]. A couple of papers, including review papers, have reported about the physicochemical, biochemical, and pharmacological characteristics of rose hydrosols. The main information is based on *R. damascena* hydrosol data [2,3,4,5,6,7,8,9,10]. The hydrosol antiseptic and antispasmodic actions have been established [6]. Hydrosol prepared from the Bulgarian *Rosa damascena* shows the ability to inhibit skin inflammation caused by *Candida albicans* and methicillin-resistant *Staphylococcus aureus* [11]. It was reported that the effect depends on the extraction method and concentration applied [12]. Rose hydrosol is effective in the prevention of fresh-cut taro browning [10]. On the contrary, other authors have not obtained an antibacterial effect of *R. damascena* hydrosol on skin flora after hand rubbing was detected [13]. Less is known about the volatile constituents of white rose (*Rosa alba* L.) water and its biological activity [8,14,15,16]. 

The yield of both essential oil and hydrosol is influenced by many factors. Genotype, harvest time, geographical region [17], method of extraction, and storage are decisive [3,18,19]. During the harvesting process, hand picking must be followed [20]. The raw material for the distillation such as leaves, petals, flowers, or fruits plays a crucial role [14,15,21]. Distillation methods are also important—factory-type distillation or village-type distillation [22]. 

Rose hydrosols contain valuable chemical compounds that are circa 10–50% of the rose oil’s constituents, including flavonoids, anthocyanins, terpenes, and glycosides. The determination and analysis of the essential oil components and their hydrosols can be performed using various methods, such as GC-MS [2,5,8,23,24] and HPLC [25,26]. For results interpretation reference samples with various origins—synthetic, conventional, and traditional samples, were used [27]. A percentage comparison between the essential oil and hydrosol of *Rosa damascena* dried petals and fresh flowers for cosmetics uses were published in a Tentative Report for Public Comment [28]. 

The chemical composition of essential oils and their byproducts, as well as the balance of ingredients, play a crucial role when interpreting their effects [29,30]. Information about the biological effects and genotoxic activity exists only for some chemical compounds. Geraniol, as an important component of rose essential oil, shows the potential to decrease cytotoxic and genotoxic action of MNNG in root tip meristem cells of *Hordeum vulgare* and human lymphocytes *in vitro* [31]. Geraniol exhibited a cytotoxic effect on various types of cancer cells and suppressed tumor proliferation [32]. Another component eugenol and its analogues were also tested for antitumor activity using different human cancer cell lines. Inhibitory activity of cell growth has been established [33]. It was obtained that eugenol significantly reduced the MNNG-induced gastric tumors by suppressing NF-κB activation and modulating the expression of NF-κB target genes. Thus, eugenol offers an immense potential in cancer chemoprevention and therapy [34]. 

There is very little evidence to date with regard to the cytotoxic and genotoxic potential of *R. alba* and *R. damascena* essential oils and the byproducts of rose oil distillation. In recent years, our research group has been working to fill this gap. In this sense, we already know much more about the cytotoxic/genotoxic activity and protective antimutagenic effect of some rose products (essential oils and wastewaters) of Bulgarian *Rosa damascena* Mill. and *Rosa alba* L. on certain groups of organisms and/or cells [30,35,36]. It is important to study whether such aromatic plant products, widely used in human practice, are safe. Since no information is available concerning the cytotoxicity and genotoxicity of rose hydrosols, we set the goal to study the cytotoxic and genotoxic/clastogenic effects of *R. alba* L. and *R. damascena* Mill. hydrosols derived during the water–steam distillation of rose oils by classical cytogenetic methods in a complex of *in vivo* and *in vitro* test systems.

## 2. Materials and Methods

### 2.1. Preparation of R. alba L. and R. damascena Mill. Hydrosols

Hydrosols were obtained from roses grown in the experimental field belonging to the Institute for Roses and Aromatic Plants, Kazanlak, harvested from 2019 and 2020. The authenticity of the rose species was confirmed by Ana Dobreva. The voucher specimens were deposited in the IBER-BAS herbarium where the number of *R. damascena* Mill. is SOM 177 768 and of *R. alba* L. is SOM 177 769, respectively. The rose flowers were subjected to water–steam distillation using semi-industrial processing line. The following parameters for distillation were used: 8 kg raw rose material for charge and hydro module 1:6, in a rate of 8–10% and duration of 150 min. at distillate temperature maintained at 28–30 °C. The hydrosol obtained from this process was redistilled at very low speed to obtain the same quantity of rose hydrosol as the charged material, using the same apparatus at the same temperature [8]. After detailed analysis, the hydrosol was stored in a refrigerator at 4 °C in dark, in sterilized containers, until further use. 

### 2.2. Chemicals

The standard experimental mutagen N-methyl-N′-nitro-N-nitrosoguanidine MNNG (50 μg/mL) (CAS-Nr.: 70-25-7) used as a positive control was provided by Fluka—AG, Buchs, Switzerland. RPMI 1640 medium was from Sigma-Aldrich, (Steinheim, Germany); fetal calf serum from Sigma-Aldrich, (Sao Paulo, Brazil); phytohemagglutinin PHA and cytochalasin-B from Sigma-Aldrich, (Jerusalem, Israel); acridine orange, KCl, and acid aceticum glaciale were purchased from Sigma-Aldrich Chemie GmbH, Merck (Steinheim, Germany). Solution of 0.9% NaCl and gentamycin were provided by Sopharmacy (Sofia, Bulgaria). 

### 2.3. Test Systems and Experimental Design

Three different test systems widely used in genotoxic screening were used to study cytotoxic/genotoxic effect of rose hydrosols applying tests for induction of chromosome aberrations (CA) and induction of micronuclei (MN).

#### 2.3.1. Plant Test System *In Vivo*

The structurally reconstructed karyotype MK14/2034 of *H. vulgare* has seven easily distinguishable chromosome pairs, which allow for easier detection of the mutagen-specific features of aberration distribution [37]. Despite these special properties, this karyotype is comparable in sensitivity to its standard karyotype—spring barley.

Presoaked barley seeds (1 h in tap water) were germinated in dark for 17 h in Petri dishes on moist filter paper at 24 °C. These seeds were treated with rose hydrosols in concentrations of 6%, 14%, and 20% for 1 and 4 h. Recovery times of 18, 21, 24, 27, and 30 h were examined. For scoring chromosome aberrations, after these recovery times, seedlings were affected with 0.025% colchicine in a saturated solution of a-bromonaphthalene (2 h) and fixed in ethanol:glacial acetic acid in ratio of 3:1. The barley root tip meristems were hydrolyzed in 1N HCl at 60 °C for 9 min, stained with Schiff’s reagent, macerated in 4% pectinase, and squashed onto clean slides. Untreated meristems were used as a negative untreated control.

For scoring MN, the barley root tip meristem cells were not treated with colchicine, and the meristems were fixed after 30 h recovery time [38].

#### 2.3.2. Animal Test System *In Vivo*

Eight-week-old male and female ICR strain albino mice (21.0 ± 1.5 g b. w.) were delivered from Slivnitza animal breeding house, Bulgarian Academy of Sciences, Sofia. Animals were transported to the Animal House Facility of Institute of Biodiversity and Ecosystem Research and were kept for several days at standard laboratory conditions—temperature 20–22 °C, photoperiod 7 am to 7 pm, and unimpeded access to typical diet and fluids for rearing laboratory animals. The ICR mice were randomly allocated in four experimental groups (eight male/eight female animals each) and kept in standard cages, isolating the control and the treatment groups to avoid cross contamination. All tested substances were given as a single treatment by i.p. injection. The following experimental groups (n = 8, 4♂ 4♀ each) were defined: Group 1. Animals injected with 11% hydrosol solution (0.01 mg/mL); Group 2. Animals injected with 20% hydrosol solution (0.01 mg/mL). Group 3: Positive control group injected with a model mutagen MNNG 50 μg/mL (0.01 mg/mL). Group 4: Untreated control group received i.p. only with 0.9% NaCl (0.01 mg/mL) under identical conditions. Throughout the experiment, experimental groups were inspected twice per day after primary i.p. compound supplementation for any kind of toxicity or unwellness indications.

The protocol for chromosome aberrations was applied for each experimental group starting at the 24th (4♂4♀) or 48th (4♂4♀) after single treatment with the respective solution [39]. To receive chromosomes in metaphase stage appropriate for cytogenetical observations, a mitotic inhibitor colchicine—0.04 mg/g b.w. was introduced i.p. an hour prior bone marrow cells insulation. For scoring of micronuclei, blood smears were prepared prior colchicine treatment. All experimental animals were humanely euthanized by diethyl ether; bone marrow cells were isolated by flushing from femoral bone and subjected to subsequent hypotonization for 15 min with 0.075 M KCl at 37 °C. The cell fixation procedure includes a solution of cold methyl alcohol: glacial acetic acid (3:1), followed by dripping on pre-cleaned and pre-cooled wet microscopic glass slides and being left to naturally dry. A solution of 5% Giemsa was used for slides staining (Sigma Diagnostic).

The rodent erythrocyte micronucleus assay, according to the regulatory requirements [40,41], was used as another method for evaluation of genotoxic or clastogenic effects of the tested hydrosols. The micronucleus assay was fulfilled following the OECD test guidance No. 474 for chemicals safety evaluation [40]. Peripheral blood samples were taken once, 48 h after initial treatment, from all dose groups. For each of the eight mice in the group, 5 μL peripheral blood was collected through the tail. The blood was diluted with 45 μL Sörensen’s phosphate buffer (pH 6.8) [42], and a drop of this solution was smeared on a slide, dried, and fixed for 10 min with absolute methanol. The smeared slides were Acridine orange (AO) stained following recommendations of Hayashi et al. (1983), using some modifications. AO (50 μL of a 1 mg/mL solution) was dropped on dry blood sample slides and spread by immediately covering the slide with coverslip glass. The analysis was performed with an Axio Scope A1—Carl Zeiss Fluorescent Microscope at 400x magnification, with FITC 495 nm excitation filter.

The experiments were performed in accordance with Bulgaria’s Directorate of Health Prevention and Humane Behavior toward Animals. Bulgarian Food Safety Agency (BFSA) published Certificate number 125 and standpoint 45/2015 for 5-year period, for use of animals in experiments for the Stephan Angeloff Institute. The Ethical Committee of the Stephan Angeloff Institute approved the experimental design and protocols of the work from 4 October 2020. This certificate was obtained in connection with a project application, and the experiments were carried out from 2020–2021.

#### 2.3.3. Human lymphocytes *In Vitro*

Lymphocyte cultures were prepared from peripheral venous blood of healthy non-smoking/non-drinking donors (men and women), who do not accept any medication, aged between 33 to 40 years. Each culture contained RPMI 1640 medium, 12% fetal calf serum, 40 mg/mL gentamycin, and 0.1% mitogen phytohemagglutinin (PHA) and was cultured at 37 °C. 

The method of Evans [43] was applied to study the chromosome aberrations. The lymphocyte cultures were treated with each rose hydrosol in concentrations of 6%, 11%, 14%, and 20% for 1 h and 4 h. After the treatment, the cells were washed in fresh RPMI medium and cultured at 37 °C. At the 72nd hour after PHA stimulation, the cells were affected with 0.02% colchicine, followed by hypotonization in 0.56% KCl and fixation in solution of methanol: acetic acid (3:1, *v*/*v*). After centrifugation, the pellet was dropped on clean glass slides and stained in 2% Giemsa. Untreated lymphocyte samples were used as a negative control.

For micronuclei at the 44th hour after PHA stimulation, cytochalasin-B (6 μg/mL) was added to each culture according to cytokinesis-block micronucleus (CBMN) method of Fenech [44]. After 24 h the lymphocytes were centrifuged, hypotonized with 0.56% KCl, and fixed in methanol: acetic acid (3:1). The suspension obtained after subsequent centrifugation was dropped onto clean slides and stained in 2% Giemsa.

All procedures were conducted corresponding to the Declaration of Helsinki, approved by Commission on Ethics and Academic Unity of Institute of Biodiversity and Ecosystem Research (Number: No 1 from 18 February 2022). All donors were informed about the experimental procedure and signed written informed consent forms.

### 2.4. Cytogenetic Endpoints

The cytotoxic effect was evaluated by mitotic index, PCE/(PCE + NCE) ratio, and nuclear division index (NDI). To assess the genotoxic/clastogenic activity of the tested rose hydrosols, induction of chromosome aberrations and induction of micronuclei were used as endpoints. 

#### 2.4.1. Endpoints for Cytotoxicity

##### Mitotic Index (MI)

The value of mitotic index, which gives information about the cytotoxic effect of the tested hydrosols, was assessed in all three test systems. The mitotic index is the number of metaphases per 1000 observed cells per each experimental variant. For animal cells, to assess the mitotic division, protocol of Darzynkiewicz [45] was used, where the MI was determined by calculating all dividing bone marrow cells among not less than 1500 counted cells per laboratory mouse. 

##### PCE/(PCE+NCE) and Nuclear Division Index (NDI)

To obtain additional information about the cytotoxic effect of the rose hydrosols, PCE/(PCE + NCE) ratio in each treated ICR mice was calculated, where PCE were the polychromatic erythrocytes and NCE—normochromatic erythrocytes. 

In human lymphocytes, the cytotoxicity of the tested substances was also assessed by nuclear division index (NDI), using test for induction of micronuclei. The following formula was used for its calculation, (N1 + 2N2 + 3N3 + 4N4)/N, where N1–N4 are the number of cells with 1–4 nuclei, and N is the total number of scored cells. 

#### 2.4.2. Endpoints for Genotoxicity

##### Induction of Chromosome Aberrations (CA)

**Figure 1:**
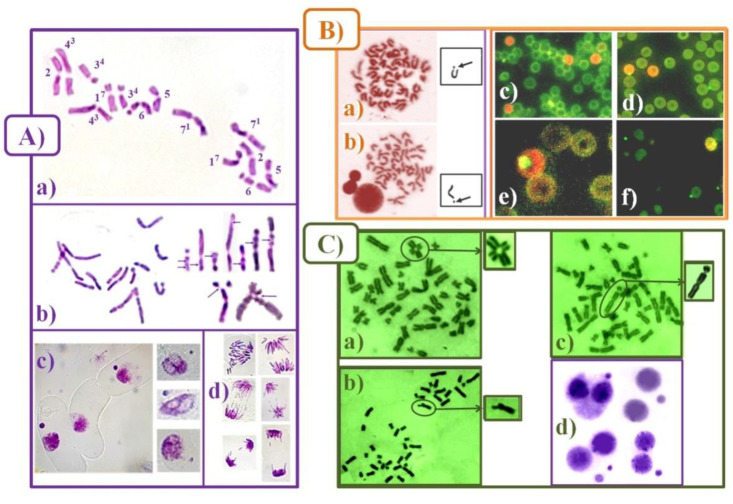
Chromosome aberrations (CA), micronuclei (MN), and mitotic disturbances detected after treatment with hydrosols derived during water–steam distillation of essential oils of Bulgarian *R. damascena* Mill. and *R. alba* L. in: (**A**) plant test system—*H. vulgare* (**a**) reconstructed karyotype, (**b**) different types of chromatid aberrations–isochromatid breaks (above), chromatid translocations (under part), (**c**) MN, and (**d**) spindle defects; (**B**) animal test system (**a**,**b**) chromosome fragments, (**c**–**f**) PC and NC erythrocytes with MN; (**C**) human lymphocyte cells (**a**) translocation, (**b**) chromatid break, (**c**) isochromatid break, and (**d**) MN.

To assess the genotoxic effect of rose hydrosols, the percentage of well-spread metaphases with chromosome aberrations (MwA% ± SD) was calculated in all test systems. Chromatid breaks, isochromatid breaks, chromatid translocations, intercalary deletions, centromere fusions, and telomere/telomere fusions were determined (Figure 1). More than 2000 cells were calculated for induction of chromosome aberrations in barley test system and human lymphocytes from each experimental variant. Up to 50 well-dispersed metaphases from either laboratory mouse were subjected to chromosomal aberrations analysis performed by light microscopy (Olympus CX 31) × 1000.

“Aberration hot spots” were determined in barley chromosomes (reconstructed barley karyotype MK14/2034) to receive detailed information about the specific clastogenic effects. This karyotype gave the opportunity to study defined chromosome segments in different chromosomal positions and their involvement in induced aberrations. The “aberration hot spots” in the plant chromosomes were determined to obtain information about the DNA segments with higher susceptibility to the tested hydrosol sample and/or to the mutagen. To analyze the regional specificity of aberration induction the metaphase chromosomes of *H. vulgare* were subdivided into 48 segments of almost equal sizes. The segments are numbered with respect to their position in the standard karyotype [37,46] (see Figure 1A).

##### Induction of Micronuclei (MN)

Generally, the micronucleus test is fast and easy to perform. Between 3000–6000 cells for the presence of MN and spindle defects, which later forms MN too for each treatment variant, were inspected for barley and human lymphocytes *in vitro* (Figure 1A(c),C(d)). Percentage of micronuclei (MN% ± SD) was calculated. 

The criteria used for the identification of micronuclei and to distinguish them from artifacts (Figure 1B(c–f)) in the cytoplasm of animal cells were described by Schmid [47]. Micronuclei were easily distinguishable in polychromatic erythrocytes (PCE), which emit red fluorescence [48]. From each slide, at least 2000 PCE or at least 4000 PCE per animal were screened for the incidence of micronucleated immature erythrocytes. The ratio of PCE to total (PCE + NCE) erythrocytes was defined for each laboratory mouse separately, by counting at least 2000 normochromatic erythrocytes per animal. Only monolayers without overlapping cells were targeted for each slide. 

In the *in vivo* animal test system, the relationship between PCE and NCE as well as the MN rates of the exposed and control samples (negative, positive) were examined.

### 2.5. Statistical Analysis

Each experiment was triplicate. One-way ANOVA with two-tailed Fisher’s exact test was used for statistical analysis of the different treatment variants for all three test systems (Microsoft Excel 2010). The statistical differences were assayed as *p* > 0.05 not significant, * *p* < 0.05 significant, ** *p* < 0.01 more significant, and *** *p* < 0.001 extremely significant.

The aberrations hot spots in the reconstructed barley karyotype were calculated followed the protocol of Rieger et al. [49] and Jovtchev et al. [46].

## 3. Results

### 3.1. Cytotoxic Effects of Rosa alba L. and Rosa damascena Mill. Hydrosols

#### 3.1.1. Mitotic Index (MI)

**Figure 2:**
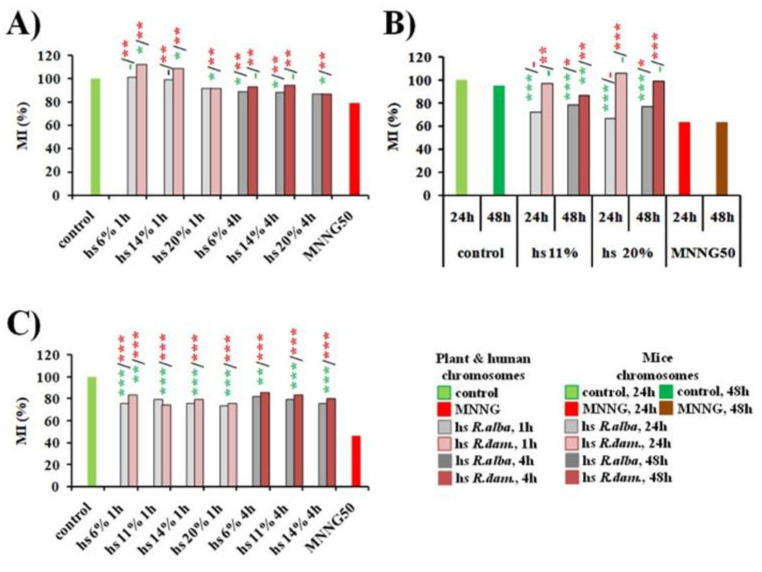
Cytotoxic activity of hydrosols (hs) derived during water–steam distillation of essential oils from *R. alba* L. and *R. damascena* Mill. assessed by the value of mitotic index (MI) in: *H. vulgare* (**A**), mice bone marrow cells (**B**), and human lymphocytes (**C**). Mitotic activity was presented as a percentage of untreated control. * *p* < 0.05, ** *p* < 0.01, *** *p* < 0.001, and non-significantly versus untreated control (before slash) versus positive control MNNG (after slash).

No or very low cytotoxic effect was calculated by MI both for hydrosol from *R. alba* L. and *R. damascena* Mill. applied at concentrations of 6–20% (for 1 and 4 h) in *H. vulgare*, compared to the untreated control (Figure 2A). A significant reduction in mitotic activity was obtained after *R. alba* hydrosol application in the bone marrow cells of all experimental animal groups (*p* < 0.05) compared to the control group. Surprisingly, the MI values obtained were close with those calculated in the experimental group treated with MNNG (50 μg/mL) (Figure 2B). In contrast to *R. alba* hydrosol, a lack of antiproliferative action of the *R. damascena* hydrosol solutions was obtained. The human lymphocytes were the most sensitive to both rose hydrosols compared to barley and mice cells. The cytotoxic effect of *R. alba* hydrosol (6%, 11%, 14%, and 20%) assessed by MI value in lymphocytes was not high and decreased in a concentration-dependent manner. The MI values calculated for *R. damascena* did not show any dependence on the concentration applied and were slightly increased with the duration of treatment. (Figure 2C). The mitotic activity observed in all three test systems after hydrosols application was much higher compared to the cytostatic action of the MNNG alkylating agent used as a positive control. Data from the *in vivo* and *in vitro* experiments are highly consistent and show that the hydrosols exhibited lower toxicity toward normal cells compared to MNNG (*p* < 0.001) (Figure 2). 

#### 3.1.2. PCE/(PCE+NCE)

**Figure 3:**
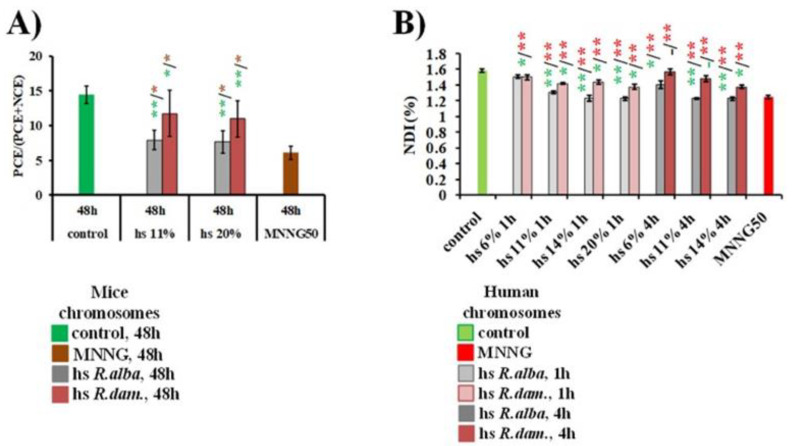
Cytotoxic activity of hydrosols (hs) derived during water–steam distillation of essential oils from *R. alba* L. and *R. damascena* Mill. evaluated by PCE/(PCE + NCE) ratio in ICR mice (**A**) and value of NDI in human lymphocytes (**B**). * *p* < 0.05, ** *p* < 0.01, and non-significantly versus untreated control (before slash) versus positive control MNNG (after slash).

The ratio showed significant differences among the *R. alba*-treated ICR mice groups (20% and 11%) and the negative control group (*p* < 0.001) in ICR mice (Figure 3A). In the presence of *R. alba* hydrosol, the percentage of PCE (7.92% ± 1.41 for 11% and 7.64% ± 1.64 for 20%) decreased significantly (*p* < 0.05; *p* < 0.01, respectively) compared with the negative control, which may indicate a suppression of bone marrow proliferation, probably due to mitotic arrest. Comparison between the positive control group with MNNG 50 μg/mL and the two hydrosol concentrations also showed a statistical significance (*p* < 0.05; *p* < 0.01), respectively. Therefore, it can be concluded that the lower, but statistically significant values, calculated after hydrosol treatment, support the assumption of a cell proliferation suppression following *R. alba* hydrosol supplementation. Similar to the mitotic index, the ratio of polychromatic erythrocytes to total number of poly- and normochromatic erythrocytes in the animal test system did not show any dose-dependent effect at the two concentrations of *R. damascena* hydrosol administered (*p* > 0.05). At an exposure of 11% and 20% *R. damascena* hydrosol solution, the corresponding calculated mean values for PCE/(PCE + NCE) (%) were 10.97% ± 2.64 and 11.75% ± 3.30, respectively. These mean values are distinguished significantly from the values in the control group (0.9% NaCl) (14.44% ± 1.27) (*p* < 0.01; *p* < 0.05, respectively). Since there was a well-expressed statistical significance compared to the data of the MNNG group (*p* < 0.001; *p* < 0.01), it can be concluded that the suppression of cell division by *R. damascena* hydrosol assessed by the ratio PCE/(PCE+NCE) is minimal.

#### 3.1.3. Nuclear Division Index (NDI)

This endpoint used as a second indicator for cytotoxic activity of the hydrosols in lymphocyte cultures was slightly reduced (*p* < 0.05, *p* < 0.01) in rose-hydrosol-treated cells compared to the negative control (Figure 3B). Surprisingly, the higher NDI values were obtained after a 4h hydrosol treatment of *R. damascena*, with values that were similar to those of the untreated control. Data obtained from the NDI showed that *R. damascena* hydrosol has low suppression activity of cell division and slightly higher than of *R. alba*.

### 3.2. Genotoxic Effects of the Rose Hydrosols

#### 3.2.1. Induction of Chromosome Aberrations

The genotoxic effect of *R. alba* and *R. damascena* hydrosols was investigated by values of metaphases with chromosome aberrations (MwA) as an endpoint.

**Figure 4:**
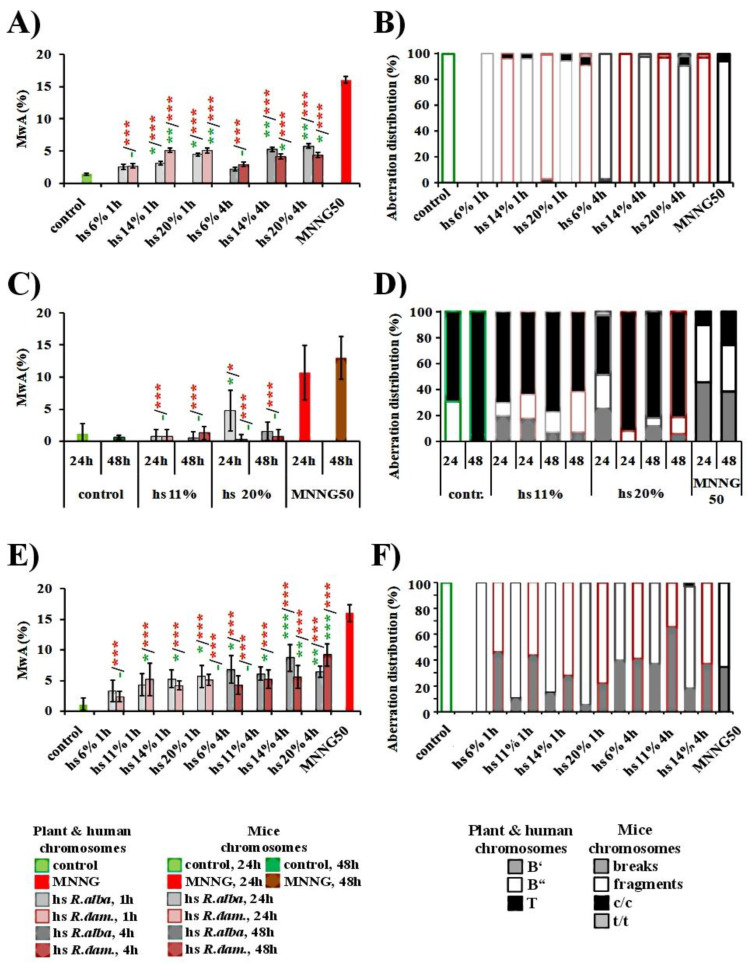
Genotoxic effect of hydrosol (hs) derived after water–steam distillation of essential oils from *R. alba* L., and *R. damascena* Mill., assessed by the frequency of metaphases with aberrations (MwA) in: *H. vulgare* (**A**), mice bone marrow cells (**C**), and human lymphocytes (**E**). Distribution of aberrations observed after treatment with both hydrosols (hs) in *H. vulgare* (**B**), mice bone marrow cells (**D**), and human lymphocytes (**F**). * *p* < 0.05, ** *p* < 0.01, *** *p* < 0.001, and non-significantly versus negative control (before slash) versus positive control MNNG (after slash).

Treatment with all rose hydrosols’ concentrations (6–20%) induced a comparatively low but statistically significant (*p* < 0.05; *p* < 0.001) genotoxic/clastogenic effect compared to the negative controls in the plant test system, animal cells, and human lymphocytes (Figure 4A,E).

The frequency of aberrations in *H. vulgare* after 1 h treatment with *R. alba* hydrosol ranged from 2.53% ± 0.4 (with 6%) to 4.47% ± 0.47 (with 20%), and treatment for 4 h induced a frequency from 2.2% ± 0.29 (with 6%) to 5.8% ± 0.32 (with 20%), respectively. Similar frequencies were observed after treatment with *R. damascena* hydrosol, namely yields between 2.73% ± 0.31 for 6% (1 h) and 5.07% ± 0.41 for 20% (1 h) and 2.93% ± 0.33 for 6% (4h), 4.40% ± 0.5% for 20% (4h) The effects of two applied concentrations of *R. alba* and *R. damascena* hydrosols (11% and 20%) on the laboratory ICR mice chromosome structures were evaluated using the bone marrow test. The data about the percentage of chromosome aberration frequencies are presented in Figure 4C. The highest percentage of aberrant mitoses (4.75% ± 3.19) was calculated in the experimental mice group, which was treated with 20% *R. alba* hydrosol at the first sampling point (24th from the beginning of the experiment), compared to other experimental variants (*p* ≤ 0.05). The clastogenic effect of the 20% hydrosol solution significantly decreases at the 48th hour (1.5% ± 1.41) and does not differ statistically from the data calculated in the 0.9% NaCl control group (1.14% ± 1.57). The results showed a considerable reduction in the percentage of mitoses with aberrations (0.75% ± 1.03 and 0.5% ± 0.92) in the bone marrow mice cells, treated with 11% hydrosol at the 24th and 48th hours compared to the data in the 20%-hydrosol-treated group at the 24th hour (*p* < 0.001). These results as well as the one obtained in the 48th hour with 20% hydrosol, are statistically undistinguishable from the data in the 0.9% NaCl control groups. The results of our experiments with laboratory ICR mice showed that only at the higher applied concentration of *R. alba* hydrosol induced a weak clastogenic effect. A slight dose-dependent effect was observed. This result attested the sensitivity of the assay to detect genotoxicity of various types of genotoxic compounds. A dose-dependent statistically significant difference was not found between the two *R. damascena* hydrosol concentrations tested. The number of aberrations did not increase significantly with the treatment period extension (at 24 and 48 h) (*p* > 0.05), as shown in Figure 4C. All mean values are quite close to the data obtained after the analysis for the untreated control group, and, as expected, there is no statistical reliability (*p* > 0.05). From the results and the performed statistical analysis, it is clear that slightly higher values for the frequency of bone marrow cells with aberrations were obtained in the experimental groups injected with the lower dose of hydrosol (11% solution).

In human lymphocyte cells, both rose hydrosols showed a statistically significant genotoxic effect (*p* < 0.05; *p* < 0.01) with the applied concentrations compared to the untreated control. A dose-dependent effect was observed after treatment with the *R. damascena* and *R. alba* hydrosols, and the frequency of chromosome aberrations was increased with the increasing of the treatment time duration. The frequency of observed chromosomal abnormalities after treatment with *R. alba* hydrosol ranged from 3.30% ± 1.70 (for 6%) to 5.70% ± 1.80 (20%) for 1h. For 4 h treatment, the obtained aberrations varied in a range from 6.8% ± 2.20 (with 6%) to 8.7% ± 2.20 (with 14%), respectively (Figure 4E). Similar effects were observed after *R. damascena* hydrosol treatment, namely yields between 2.40% ± 0.9 for 6% 1h, 5.10% ± 0.9 for 20% 1h, 4.30% ± 1.5 for 6% 4 h, and 9.2% ± 1.8 for 14% 4 h (Figure 4E). Both rose hydrosols had a similar genotoxic effect.

The clastogenic activity of both rose hydrosols at all tested concentrations was far lower (*p* < 0.001) than that of the direct mutagen MNNG (50 μg/mL) in all three test- systems (Figure 4). MNNG produced a statistically significant increase in the number of damaged cells. 

In summary, there was no or a slight dependence of treatment duration and the concentrations applied, with respect to the values of induced aberrations in barley as well as in human lymphocytes (Figure 4A,E). Human lymphocytes *in vitro* were more sensitive to both hydrosols than the two *in vivo* test systems (plant and animal cells), using chromosome aberrations as the endpoint for genotoxicity.

The spectrum of the induced chromosome aberrations in *H. vulgare* by different concentrations of *R. alba* and *R. damascena* hydrosol was mainly of isochromatid breaks, a small number of chromatid breaks, translocations, and intercalary deletions (Figure 4B). In mice bone marrow cells, predominantly centromere–centromeric fusions followed by breaks, fragments, and a very small number of telomere-to-telomere fusions were detected (Figure 4D), while, in human lymphocytes, predominantly isochromatid breaks followed by chromatid breaks were observed (Figure 4F).

**Figure 5:**
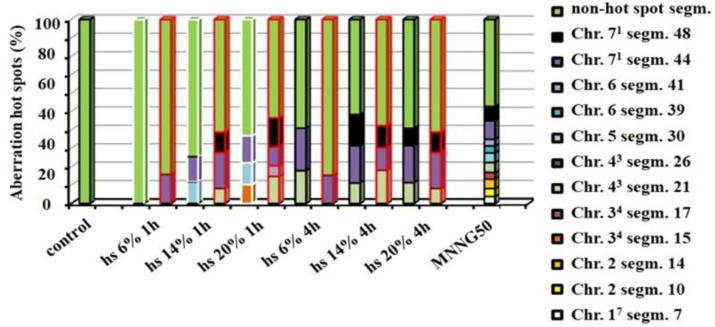
Aberration hot spots observed in the reconstructed barley karyotype MK14/2034* after treatment with different concentrations of *R. alba* and *R. damascena* hydrosols. * Reconstructed karyotype of *H. vulgare* (MK 14/2034) is a result of the combination of two simple reciprocal translocations between parts of chromosomes 1 and 7 and chromosomes 3 and 4.

The aberration hot spots in barley reconstructed karyotype induced by different concentrations of *R. alba* hydrosol showed to be dose-dependent. The lower concentrations have one aberration hot spot, the higher have up to three. The most aberration hot spots were observed in the telomere and centromere regions. The yields of aberration hot spots induced by different concentrations of both hydrosols are significantly lower than the number of hot spots induced by MNNG. As for the positive control, MNNG treatment, 11 aberration hot spots were detected (Figure 5).

#### 3.2.2. Induction of Micronuclei

**Figure 6:**
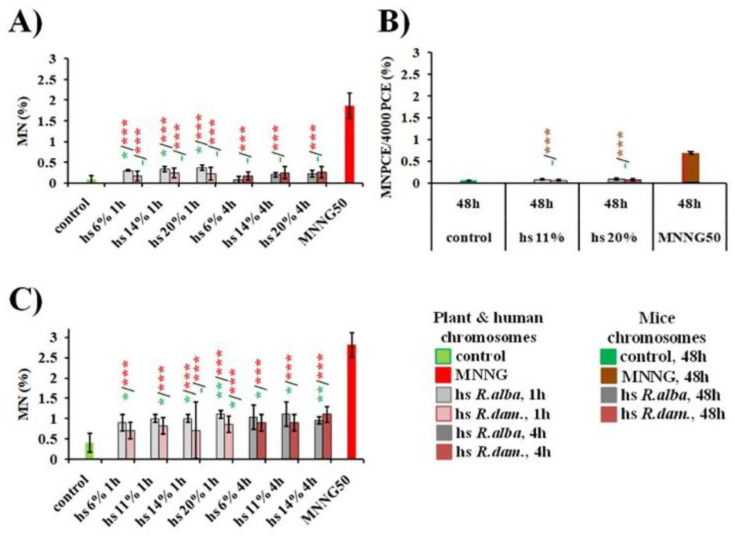
Genotoxic effect of the hydrosols (hs) derived during water–steam distillation of essential oils from *R. alba* L. and *R. damascena* Mill., assessed by the value of induced micronuclei in: *H. vulgare* (**A**), mice bone marrow cells (**B**), and human lymphocytes (**C**). * *p* < 0.05, ** *p* < 0.01, *** *p* < 0.001, and non-significantly versus untreated control (before slash) versus positive control MNNG (after slash).

In the *H. vulgare* test system, the micronuclei induced after treatment with *R. alba* hydrosol ranged from 0.30% ± 0.02 for 6%/1 h to 0.37% ± 0.06 for 20%/1 h. After 4h of treatment, an induction of MN, ranging from 0.08% ± 0.08 (6%) to 0.23% ± 0.08 (20%), was detected (Figure 6A). No clear concentration and treatment-duration dependence were observed. The data for *R. damascena* hydrosol were very close to those for *R. alba* hydrosol, namely 0.17% ± 0.11 for 6% 1h, 0.22% ± 0.16 for 20% 1h, 0.17% ± 0.10 for 6% 4 h, and 0.27% ± 0.14 for 20% 4 h (Figure 6A).

In the *in vivo* animal test system, the number of micronucleated erythrocytes MNPCE observed after treatment with *R. alba* hydrosol was given separately for each treatment group. The results of the micronucleus assay with peripheral blood erythrocytes in laboratory mice are summarized in Figure 6B. The higher frequency of MNPCE was observed in the experimental group, treated with 20% *R. alba* hydrosol (0.09% ± 0.02). The frequencies of MN in PCE were statistically undistinguishable and the results were not dose-dependent between the two hydrosol groups treated with *R. alba* (*p* > 0.001). *R. alba* hydrosol at concentration of 20% significantly increased the frequency of MNPCE from 0.06% ± 0.01 (mean value for the positive control group) to 0.09% ± 0.02 (*p* < 0.01). A significant rise in the frequency of MNPCE compared to the control was also noted following injection of 11% *R. alba* hydrosol (0.08 MNPCE/4000 PCE, *p* < 0.01). It is evident from the results of the rodent erythrocyte MN assay that *R. alba* hydrosol possesses low genotoxic activity, as it produced a slight-but-significant increase in the number of PCE with MN compared to the control group.

The data are far from the values obtained in the experimental animal group, treated with 50 μg/mL MNNG (0.69% ± 0.03). This can be seen as evidence that, despite the statistically positive result in the micronucleus test, *R. alba* hydrosol at the tested concentrations cannot be considered as genotoxic (Figure 6B).

After treatment with *R. damascena* hydrosol, a higher frequency of micronucleated erythrocytes (MNPCE) was observed in the animal group treated with 20% solution (0.07% ± 0.03) compared to that with 11%. The frequency slightly reduced from this value but was not significantly distinct from the control (*p* > 0.05), actually returning to the control level at 11% dose 48 h post-exposure (0.06% ± 0.02). As compared to MNNG (50 μg/mL (0.01 mg/mL), a significant decrease in the MN frequency (*p* < 0.001) was reported for both hydrosol solution concentrations (Figure 6B).

Our micronuclei analysis study on mice peripheral blood erythrocytes has revealed that *R. damascena* hydrosol solution, based on the concentration and time intervals analyzed, does not cause a variation in chromosome structure but, rather, a slight cytotoxic effect (a weak cytotoxicity) (Figure 6B). 

In human lymphocyte cultures, all concentrations (6–20%) of *R. alba* hydrosol applied at both time duration treatments (1 h and 4 h) showed close values of MN (0.09% ± 0.02 for 6% to 1.1% ± 0.01 for 20% 1 h treatment and 1.04% ± 0.03 for 6% to 0.96% ± 0.09 for 14% 4 h treatment). No dose- or duration-dependence were obtained among the frequency of MN after treatment with all concentrations of hydrosol in lymphocyte cells (Figure 6C). A mild statistically significant genotoxic effect was observed for the tested hydrosol concentrations, where the frequency of MN was slightly increased (*p* < 0.05, *p* < 0.01) compared to the control. The same effect was detected also for *R. damascena* hydrosol concentrations. *R. alba* and *R. damascena* hydrosol were not found to have a dose dependence (0.70% ± 0.20 with 6% for 1 h to 0.86% ± 0.20 with 20% for 1 h and 0.90% ± 0.2 with 6% for 4 h to 1.1% ± 0.20 with 14% for 4 h) for the value of MN induced by all tested hydrosol concentrations. The frequencies of MN did not increase significantly with the treatment period (1 and 4 h), as shown in Figure 6C. *R. alba* showed slightly higher genotoxic effect compared with that of *R. damascena* hydrosol assessed by MN induction, but the effect was not significantly proven. 

The genotoxic action (expressed by MN) of both rose hydrosols at all tested concentrations was far below than the values obtained for the MNNG (50 μg/mL) (*p* < 0.001) (Figure 6). Our data showed that the genotoxic effect of both hydrosols was most pronounced in human lymphocytes *in vitro*.

## 4. Discussion

Applying a complex of cytogenetical tests by various endpoints in three different experimental test systems (*in vivo* and *in vitro*), we performed a good assessment of the cytotoxic and genotoxic activities of *R. alba* and *R. damascena* hydrosols. Here, we obtained useful information about the cellular sensitivity and the effect on hereditary material resulting from byproducts application. The use of different test systems with a range of methods makes the evaluation of the effect more representative and useful.

The analysis of the chemical composition of the rose hydrosols is essential for the interpretation of the results obtained. Detailed phytochemical analysis of the *R. alba* L. and *R. damascena* Mill. hydrosols given previously showed variances in the main groups of constituents [8]. There were subtle differences, but they could explain some impact on the effects. The main differences between both hydrosols were in the content of the oxygenated monoterpenes (OM)—76.63% for *R. alba* and 65.87% for *R. damascena*. Geraniol, citronellol, and linalool belong to this group. Monoterpenoid geraniol, presented as trans-geraniol 36.44% and cis-geraniol 6.11%, is the main constituent of *R. alba* hydrosol. *R. damascena* byproduct contained almost 15% less geraniol viz 16.44% trans-geraniol and 10.81% cis-geraniol [8]. β-Citronellol was found to be the main ingredient in *R. damascena* hydrosol, namely 28.7%. This compound was with equal quantity in both rose hydrosols. Another important compound, which belongs to the group of benzenoid compounds (BC), is phenylethyl alcohol. This component is known to vary greatly under certain conditions (time of harvest and degree of freshness of the raw material). Here, the content was very close in both hydrosols, but in *R. alba* hydrosol its amount is slightly higher, 5.95%,compared to 4.95% in *R. damascena*.

Our cytogenetic analysis showed that the hydrosol of *R. alba* slightly reduces the value of MI in *H. vulgare* root tip meristem cells, whereas the tested concentrations of *R. damscena* hydrosol enhanced the cell division. On the other hand, mitotic activity/cell division was significantly reduced in mice bone marrow cells and human lymphocytes *in vitro* after *R. alba* administration. In contrast, no significant alterations of cell division in mice cells and only a weak suppressive affect were observed in human lymphocytes *in vitro* after administration of *R. damascena* hydrosol. 

Another endpoint that also provides information about the cytotoxicity, the PCE/(PCE + NCE) ratio in red blood cells of ICR mice, indicates alterations in erythropoiesis in the animal test system after application of hydrosols. *R. damascena* hydrosol reduced this ratio in much weaker extend than that of *R. alba*.

According to our data obtained for all endpoints of cytotoxicity in the three test systems, *R. alba* hydrosol possesses a higher cytotoxic effect than that of *R. damascena*. The scientific literature lacks any information on the cytotoxicity of *R. alba* hydrosol, and there are scarce data on the effect of *R. damascena*. The results obtained by us about *R. damascena* hydrosol are partially consistent with data published by Zamiri et al. [50]. According to these authors, *R. damascena* ethanol extract exhibited cytotoxicity toward the HeLa cell line. Another study also indicated that *R. damascena* and its constituents possess antitumor and anticancerogenic activities [51]. Significantly high cytotoxicity against NB4 and MCF7 cell lines was also demonstrated by Gao et al. [52], applying isolates from the flowers of *Rosa damascena*. 

Since rose petals contain some compounds, which exhibit anti-proliferative activity [53], our results, detecting moderate mitotic suppression, provide their explanation. Dose-dependent anti-proliferative activity was reported by Wedler et al. [52] in immortalized human keratinocytes with a half-maximal inhibitory concentration (IC50) of 9.78 μg/mL of polyphenol-enriched fraction of *R. damascena* Mill. Our results concerning the anti-proliferative effects of *R. alba* hydrosol might also be due to the higher quantity of such compounds. Georgieva et al. [8] reported that the content of total phenolic compounds (TPC) of *R. alba* hydrosol is more than two times higher than that of *R. damascena* hydrosol. Carnesecchi et al. [54,55] reported anti-proliferative activity of monoterpenoid geraniol in cancer cells using various assays. More recent studies also identify powerful cytotoxicity of geraniol over tumor cells [56,57,58]. Our previous study with geraniol also showed its cytotoxic effect on human lymphocytes *in vitro*, which was dependent on the concentration used [31]. It is interesting to note that geraniol in combination with citronellol exhibit some toxic effects on mosquito species *Cx. pipiens* adults, and these compounds are more toxic compared to linalool alone [59]. Another main chemical component of hydrosols, namely phenylethyl alcohol, has demonstrated variation in the developmental toxicity in rats and skin toxicity in humans [60]. These studies showed that the cytotoxic effect of hydrosol clearly depends on the chemical content of the tested by-product, treatment scheme and target organisms. The low toxicity and/or negative results obtained by us in the *in vivo* test systems suggest biotransformation and excretion of the chemical components of the hydrosols by the organism. These factors have been proposed to calculate the realistic safety margin of phenylethyl alcohol for various organisms [60]. 

Analysis of the genotoxic activity of the two rose hydrosols showed that the value of the induced chromosomal aberrations also depended on the type of rose hydrosols. The yields of structural chromosomal damages increased after treatment with both hydrosols, especially with the *R. alba* hydrosol in laboratory ICR mice and in human lymphocytes *in vitro*. The results from CA showed that *R. damascena* hydrosol failed to elicit significant chromosome anomalies at the tested dose and/or duration of treatment. The present study detected an increase also the micronuclei frequency by both hydrosols in plant cells and cultured human lymphocytes as well as the micronucleated polychromatic erythrocytes in animal cells compared with the non-treated cells. The assessment by this endpoint for genotoxicity also demonstrated lower DNA damage induced by *R. damascena* compared to that by *R. alba* hydrosol. The genotoxic effect observed by us depends on a complex of conditions such as the target cells and the treatment scheme. Human lymphocytes *in vitro* were the most sensitive to both hydrosols, followed by higher plant and animal cells. The lower genotoxic effect of rose hydrosols in the *in vivo* test system for laboratory mice, compared with that in human lymphocytes *in vitro*, is probably due to intact metabolic processes, such as the degradation and excretion of the main hydrosol compounds. In the *in vitro* test system an instantaneous genotoxic effect is registered, without metabolic biotransformation processes. On the other hand, the cell wall in plants makes it more difficult for genotoxins to enter the cell and induce DNA damage. 

The combinations of chemical components and their amount in the byproducts are probably of a great importance for the genotoxic activity of the tested hydrosols. The TPC content is reported to depend on the plant raw material (leaves, fruits, flowers, etc.) or the product (oil, hydrosol, wastewater, etc.) [61]. The higher quantity of TPC in *R. alba* probably affects not only the cytotoxicity of the hydrosol but also the observed higher genotoxicity compared to that of *R. damascena*, especially in human lymphocytes *in vitro*. Our study on the effect of monoterpenoid geraniol (10–100 μg/mL) did not show a high genotoxic effect in human lymphocytes *in vitro* and showed a weak one in higher plants [31]. Other authors also reported that geraniol applied in CHO cells at concentrations of 78.1 and 156.3 g/mL, in presence of the S9 fraction, significantly increased the number of cells with structural aberrations. On the other hand, no statistically significant enhancement in the frequency of PCE with micronuclei in bone marrow of mice treated with high concentrations of geraniol was detected [62]. 

The lower amount of TPC and probably BC suggests a lower or no genotoxic effect of the *R. damascena* hydrosol. Consequently, in the present studies, no or a low clastogenic or aneugenic effect was obtained in the treatment regimen in bone marrow cells, and a low genotoxicity in barley was obtained after application of hydrosol to *R. damascena*. Our findings with the plant test system *in vivo* and animal test system *in vivo* reciprocate earlier investigations reporting a lack of chromosome damage [62,63,64] in various chemopreventive herbal extracts and phytochemical studies. Since cytogenetic results for geraniol, which together with citronellol are the main constituents of the *R. alba* and *R. damascena* hydrosols, are lacking in the literature, our results are expected and logical.

In our study, we have not observed high positive results for either rose hydrosols in the *in vitro* assays with human lymphocytes, and the consistent negative results or low cytotoxic/genotoxic effects observed in more relevant *in vivo* test systems using chromosomal aberrations and micronucleus analysis suggest that *R. damascena* and *R. alba* hydrosols possess low genotoxic risk to humans when used.

## 5. Conclusions

This study presents an evaluation of the cytotoxic/genotoxic effects of hydrosols obtained after water–steam distillation of essential oils from Bulgarian *R. alba* L. and *R. damascene* Mill. using tests for genotoxicity in three different *in vivo* and *in vitro* test systems. 

The following conclusions can be drawn.

The *R. alba* hydrosol does not show any cytotoxic effect in the plant test system *in vivo*. A comparatively low cytotoxicity was observed in mice bone marrow cells and human lymphocytes *in vitro* at all concentrations compared to the untreated control. It exhibited a low, concentration- and time-dependent, statistically significant genotoxic/clastogenic effect compared to untreated controls, as assessed by chromosomal aberrations and micronuclei in all test systems.

The *R. damascena* hydrosol exhibited weak cytotoxic and clastogenic effects at all concentrations applied in both the *in vivo* and *in vitro* test systems. The observed effect does not depend on the duration of treatment in all three test systems.

The results, obtained here, show that the hydrosols did not possess high cytotoxic and genotoxic activities. The effects depend on the type of rose hydrosols, the concentrations applied, and the sensitivity of the test system used. Both rose hydrosols have the potential to become a part of therapeutic applications. Furthermore, these products can be used for cosmetic purposes and in the food industry. The sensitivity of certain cell types must be considered.

## Data Availability

All the obtained data of this research are presented in the manuscript.
